# NF-κB inducible miR-30b-5p aggravates joint pain and loss of articular cartilage via targeting SIRT1-FoxO3a-mediated NLRP3 inflammasome

**DOI:** 10.18632/aging.203466

**Published:** 2021-08-29

**Authors:** Haiting Xu, Jie Zhang, Xiaoming Shi, Xiaoyang Li, Chao Zheng

**Affiliations:** 1 Department of Hand and Plastic Surgery, The Second Affiliated Hospital and Yuying Children’s Hospital of Wenzhou Medical University, Wenzhou 325027, Zhejiang, China; 2 Department of Stomatology, Linyi People’s Hospital, Linyi 276003, Shandong, China; 3 Department of Reparative and Reconstructive Surgery, Linyi People’s Hospital, Linyi 276003, Shandong, China

**Keywords:** osteoarthritis, inflammation, miR-30b-5p, silent information regulator 2 homolog 1, NLRP3

## Abstract

MicroRNAs (miRNAs) contribute to osteoarthritis (OA) development. Nevertheless, the function and mechanism of miR-30b-5p in OA are unclear. In the present article, we gauged the miR-30b-5p level in OA patients and analyzed its correlation with OA stages. Then, we conducted *in-vivo* and *in-vitro* gain-of-function assays to determine the function of miR-30b-5p, silent information regulator 2 homolog 1 (SIRT1) and Fox. Cell counting Kit-8 (CCK-8) assay, BrdU assay and flow cytometry were utilized to gauge cell viability and apoptosis of human chondrocyte (HC-A). The targeting association between miR-30b-5p and SIRT1 was validated through the dual-luciferase reporter assay and RNA immunoprecipitation (RIP) experiment. The results signified that miR-30b-5p was up-regulated in OA patients, OA rats and interleukin-1β (IL-1β)-induced chondrocytes. The higher miR-30b-5p expression brought about progressive stages of OA patients and enhanced levels of pro-inflammatory mediators in the synovial fluid. Functionally, overexpressing miR-30b-5p hampered cell viability, aggravated chondrocyte apoptosis and NLRP3 inflammasome activation induced by IL-1β, while down-regulating miR-30b-5p exerted the reverse effects. The *in-vivo* experiment exhibited that down-regulating miR-30b-5p improved joint pain and loss of articular cartilage in the rats with restrained inflammation and NLRP3 inflammasome activation. Mechanistically, miR-30b-5p targeted the 3’-non-translated region (3’UTR) of SIRT1, and miR-30b-5p was inducible with NF-κB phosphorylation enhancement. Overexpressing SIRT1 or inhibiting NF-κB relieved miR-30b-5p-induced apoptosis and NLRP3 inflammasome activation by promoting FoxO3a, while down-regulating SIRT1 or FoxO3a reversed miR-30b-5p-in-induced anti-inflammatory and apoptosis-suppressive effects. Collectively, NF-κB-induced miR-30b-5p modulates chondrocyte apoptosis and OA progression by regulating the SIRT1-FoxO3a-mediated NLRP3 inflammasome.

## INTRODUCTION

Osteoarthritis (OA) is a chronic arthropathy associated with articular degeneration, manifested as articular cartilage degeneration and bone hyperplasia, often found in the elderly [1, 2]. OA is usually manifested as joint pain and poor movement, and subchondral ossification, trabecular fracture, and cystic changes are shown under X-ray examination [3]. In recent years, the OA incidence rate is on the rise due to population aging. Studies have stated that multiple inflammatory factors, such as TNF-α, IL-6 and IL-1β, contribute to OA development. Notably, the elevation of IL-1β facilitates the profiles of cyclooxygenase-2 (COX-2) and nitric oxide synthase (iNOS), thus expediting OA development [4]. Hence, it is crucial to inquiry into the specific mechanism of OA and reduce the inflammatory mediator release in OA treatment.

MicroRNAs (miRNAs) are noncoding single-stranded RNAs that are 18-25 nucleotides long. They regulate tumor progression, inflammation, angiogenesis, and so on. Meanwhile, miRNAs are implicated in OA evolvement. For instance, several studies have demonstrated that miRNA-34a induces synovium cell apoptosis in OA by down-regulating TGIF2 [5]. Additionally, Li F et al. indicated that miRNA-103 enhances chondrocyte apoptosis and accelerates the OA process by reducing the SPHK1 activity and weakening the PI3K/AKT activation [6]. miR-30b-5p is a vital miRNA. Previous researches have claimed that NF-κB-mediated miR-30b regulation plays a key role in Ang II-mediated cardiomyocytes targeting Bcl-2 [7]. Meanwhile, Yang L et al. have illustrated that cannabinoid receptor 1 (CB1) regulates the NLRP3 profile and the NLPR3 inflammasome activation in macrophages by regulating the miR-30b-5p axis, thereby easing liver inflammation [8]. Nevertheless, the action of miR-30b-5p in OA has not yet been discussed.

Silent information regulator 2 homolog 1 (SIRT1) is a nicotinamide adenine dinucleotide (NAD)-dependent deacetylase that modulates various pathways by deacetylating transcription factors. FoxO3a is the most significant member of the mammalian family of forkhead transcription factors [9, 10]. Studies have shown that miRNA-34a influences chondrocyte apoptosis and proliferation in OP pathogenesis by targeting SIRT1/p53 [11]. Besides, inhibiting miR-301A eases LPS-induced chondrocyte injury by up-regulating SIRT1 and activating the PI3K/AKT and NF-κB pathways [12]. Bai Y et al. also showed that miR-122 exerts an essential function in the extracellular matrix (ECM) degradation of OA chondrocytes by regulating the SIRT1 axis [13]. Nevertheless, whether miR-30b-5p contributes to OA by regulating SIRT1 and its transcription factor FoxO3a remains elusive.

Nod-like receptor family, pyrin domain containing 3 (NLRP3) inflammasome pertain to the NLR family, which is formed by NLRP3 scaffold, apoptosis-associated speck-like protein (ASC) and Caspase-1, and acts as an activating factor in the Caspase-1 pathway [14]. Increasing studies have demonstrated that NLRP3 inflammasomes produce pro-inflammatory cytokines and degrading enzymes like matrix metalloproteinase 3 (MMP-3), leading to cartilage degeneration and synovial inflammation [15]. Repressing NLRP3 inflammasome makes much sense in treating OA. For example, Dong HC et al. have stated that miR-223-3p directly targets NLRP3, and overexpressing miR-223-3p restrains IL-1β-induced chondrocyte apoptosis and inflammation, and overexpressing miR-223-3p restrains IL-1β-induced chondrocyte apoptosis and inflammation [16]. Meanwhile, triptolide attenuates the malignant progression of OA by regulating miR-20b/NLRP3 [17]. However, whether miR-30b-5p enhances the role of NLRP3 inflammasome in the inflammatory progression of OA remains to be further explored.

Here, we discovered the miR-30b-5p profile in OA patients’ joint fluid was significantly increased, and overexpressing miR-30b-5p aggravated chondrocyte apoptosis and inflammation *in vitro*. Therefore, both *in-vitro* and *in-vivo* experiments were implemented to probe the impact of miR-30b-5p on articular cartilage apoptosis in OA rats and its specific mechanism, thus providing some references for clinical research and treatment of OA from the molecular level.

## MATERIALS AND METHODS

### Collection of patient tissues and specimens

Cancerous tissues of 40 OA patients (17 females and 23 males, 55~65 years old) and matched normal samples from uninjured areas in 15 healthy donors were harvested from their knee joints by surgery in Linyi People’s Hospital and preserved at -80° C. All patients signed informed consent forms, and all experiments were authorized by the Research Ethics Committee of Linyi People’s Hospital and following the Declaration of Helsinki and institutional guidelines.

### Establishment of the OA model in SD rats

All animal treatments were authorized by the Animal Care and Use Committee of Linyi People’s Hospital. Forty ten-week-old male SD rats (Shanghai SLAC Laboratory Animal Co., Ltd.) were randomized into four groups: the sham group, the OA negative control (OA+NC-in) group, the OA miR-30b-5p inhibitor group (OA+ miR-30b-5p-in), and the OA miR-30b-5p mimic group (OA+miR-30b-5p). The SD rat OA model was stimulated through surgical DMM [18, 19]. Briefly, the rat’s right knee capsule in the OA+NC-in group, OA+ miR-30b-5p-in group and OA+ miR-30b-5p group was cut off from the tendon of the medial patella. The medial meniscus ligament was cut open with microsurgical scissors. In the sham group, the joint capsule was incised while the medial meniscus ligament was not damaged. The rats could eat and drink freely and were fed at 25±2° C with 50%±15% humidity, with 12 hours of light/dark cycle. The rats in the OA+NC-in group and OA+miR-30b-5p-in group were injected with 800 pmol of miR-30b-5p negative control or miR-30b-5p antagomir 6 hours before the surgery, and nine rats in each group were optionally selected and executed. Rat knees were collected for histopathological analysis for the following tests.

### Cell culture and transfection

Human chondrocytes HC-A were bought from the Cell Center of the Chinese Academy of Sciences (Shanghai, China). Cells were grown in the RPMI1640 medium containing 10% fetal bovine serum (FBS) and 1% penicillin/streptomycin (Invitrogen, Carlsbad, CA, USA) at 37° C with 5% CO_2_. RPMI1640 and FBS were obtained from Thermo Fisher Scientific (Waltham, MA, USA). In the logarithmic growth phase, 0.25% trypsin (Thermo Fisher HyClone, Logan, UT, USA) was adopted for trypsinization and sub-culture. As reported previously, IL-1β was applied to induce the OA chondrocyte model [20]. HC-A cell lines were treated with 5.0 ng/mL exogenous recombinant human IL-1β (R&D Systems, Inc.) for 3, 6, 12, and 24 hours, respectively.

SIRT1 overexpression plasmid (SIRT1) and its corresponding negative control (vector), small inference RNA (si-RNA) SIRT1 (si-SIRT1), si-FoxO3a, miR-30b-5p mimics, miR-30b-5p inhibitors (miR-30b-5p-in), and the negative controls (si-NC and miR-NC) were constructed and synthesized by Guangzhou FulenGen Co., Ltd., China The NF-κB inhibitor BAY 11-7082 (Article No. HY-13453) was bought from MedChemExpress (Monmouth Junction, NJ, USA). HC-A cells in the logarithmic growth stage were inoculated in 24-well plates (5×10^5^/well). When the cell growth was stable, Lipofectamine 2000 (Invitrogen, Shanghai, China) was applied to transfect the above expression vectors into the cells. After 24 hours, the primary medium was substituted by a fresh and complete medium. The culture was continued for 48 hours at 37° C with 5% CO_2_, and the profiles of miR-30b-5p, SIRT1 and FoxO3a were compared by quantitative reverse transcription-polymerase chain reaction (RT-qPCR) or western blot (WB) to verify the transfection efficiency.

### RT-qPCR

Total RNA was separated from tissues or cells with the TRIzol reagent (Invitrogen, Waltham, MA, USA). Nanodrop-spectrophotometer determined RNA concentration and purity. Then, the PrimeScRIPt-RT Kit (Promega, Madison, WI, USA) was utilized to transcribe 1 μg of RNA into DNA. Next, SYBR®Premix-Ex-Taq™ (Takara, TX, USA) and ABI7300 were employed for RT-qPCR. The total volume of the PCR system was 30 μL and 300 ng of cDNA was included in each sample. The amplification was performed with an initial denaturation at 95° C for 10 minutes, with 45 cycles. All fluorescence data were quantified relatively. U6 was the endogenous control of miR-30b-5p, and GAPDH was that of SIRT1, IL-1β, IL-6, TNF-α, IL-18, and FoxO3a. RT-qPCR was conducted in triplicate. Guangzhou Ribo Biotechnology Co., Ltd., China synthesized the primers (Table 1).

**Table 1:** Primer sequences of each gene.

**Gene name**	**Primer sequence (5`→3`)**
miR-30b-5p	forward: CAGTGCAGGGTCCGAGGT
	reverse: AAGCGCCTTGTAAACATCCTACA
FOXO3A	forward: AGCCAGTCTATGCAAACCCT
	reverse: CCAACCCATCAGCATCCATG
SIRT1	forward: TATGCTCGCCTTGCTGTAGA
	reverse: AACCTGTTCCAGCGTGTCTA
TNF-α	forward: CAGGGGCCACCACGCTCTTC
	reverse: CTTGGGGCAGGGGCTCTTGA
IL-6	forward: ATGAACTCCTTCTCCACAAGCGC
	reverse: GAAGAGCCCTCAGGCTGGACTG
IL-1β	forward: TCCCTTCATCTTTGAAGAAGA
	reverse: GAGGCCCCAAGGCCACAGG
IL-18	forward: TTCAAGACCAGCCTGACCAA
	reverse: GCTCACCACAACCTCTACCT
GAPDH	forward: TGATCTTCATGGTCGACGGT
	reverse: CCACGAGACCACCACCTACAACT
U6	forward: CTCGCTTCGGCAGCACA
	reverse: AACGCTTCACGAATTTGCGT

### Western blot (WB)

HC-A cells or the cartilage tissue of rats were harvested, rinsed with cold PBS three times, and added to 100~200 μL RIPA lysate (Beyotime Biotechnology, Shanghai, China). The cells underwent ultrasonic water-splitting, and the protein content was quantified by the Bradford method. The same amount of protein in each group was subjected to 10% SDS-PAGE and then transferred to PVDF membranes (Millipore, Bedford, MA, USA). Afterward, the primary antibodies (1:1000) of Bax (ab32503), cleaved-Caspase3 (ab2302), NLRP3 (ab214185), ASC (ab180799), cleaved Caspase-1 (ab74279), MMP3 (ab52915), MMP13 (ab39012), SIRT1 (ab189494), FoxO3a (ab109629), NF-ĸB p65 (ab207297), p-NF-ĸB p65 (ab239882), and GAPDH (ab181602) were added and incubated at 4° C overnight. After the membranes were cleaned twice with TBST, they were incubated at room temperature for 1 hour with fluorescein-labeled Goat anti Rabbit IgG (ab205718,1:2500). The above antibodies were obtained from Abcam (Cambridge, UK). At last, the membranes were rinsed three times, exposed with enhanced chemiluminescence (ECL) chromogenic agent (Millipore, Bedford, MA, USA), and captured with a membrane scanner.

### Cell counting kit-8 (CCK8) method

In the logarithmic growth stage, HC-A cells were trypsinized (2×10^3^ cells/mL) and inoculated in 96-well plates (100 μL suspension/well), and each group had three duplicated wells. The 96-well plates were then further incubated. After 24 hours, 10 μL CCK8 solution (Beyotime Biotechnology, Shanghai, China) was added to each well and incubated for 1 hour. After that, a microplate reader was utilized to review the optical density (OD value) of each well (450 nm wavelength) at 24, 48, 72, and 96 hours.

### Flow cytometry

After different treatments, HC-A cells were trypsinized and harvested via centrifugation (1500 rpm, 3 min). The apoptosis detection kit (Shanghai Zeye Biotechnology Co., Ltd., China, article number: ZY140626) was utilized to process the collected cells. After rinsing cells with PBS twice, we added 400 μL pre-cooled PBS, 10 μL AnnexinV-FITC, and 5 μL PI, respectively. The cells underwent incubation at 4° C 30 min away from light, and then flow cytometry was employed for apoptosis assessment. The apoptotic cell percentage was calculated by computer software.

### Safranin O-fast green staining

The knee joints were put in 4% poly formaldehyde and immobilized for 24 hours. After the joints were conventionally dehydrated, transparentized and paraffin-embedded, they were sectioned (5 μM). The Safranin O/Fast Green staining kit (ICH World, Woodstock, MD, USA) was applied for Safranin O-fast green staining following the manufacturer's guidelines. Quantification of Safranin O-positive cartilage area and thickness was made, and Image-Pro Plus 6.0 (Media Cybernetics, Bethesda, MD, USA) was employed for histomorphometric analysis.

### Haematoxylin-eosin (HE) staining and immunohistochemistry

Cartilage blocks and subchondral bones were cut into 1.0cm × 1.0cm × 0.5cm pieces. Then, the pieces were fixed with neutral formalin solution for three days, decalcified with 30% formic acid solution for 14 days, and dehydrated with gradient ethanol. After paraffin embedding, the pieces were sectioned (5 μM). The cartilage specimens were dewaxed, hydrated, and dyed with Harris alum hematoxylin (Fuzhou Maixin Biotechnology, China) for 5 min. After being cleaned in 0.5% hydrochloric acid alcohol for 10 s and dyed with eosin for 40 s, the specimens were dehydrated, transparentized, fixed with neutral balsam, and reviewed under a microscope. The chondrocyte nucleus was blue and other tissues were pink. For detecting p-NF-κB expression in the cartilage, immunohistochemistry was carried out according to the previous study [21]. The primary antibody was the Anti-Rabbit NF-kB p65 (phospho S276) antibody (ab194726, Abcam) and the secondary antibody is Goat Anti-Rabbit IgG H&L (HRP) (ab205718, Abcam). Finally, the histopathological changes were observed under a light microscope.

### Dual-luciferase reporter assay

pGL3-SIRT1-wild Type (SIRT1-WT) and pGL3-SIRT1-mutant (SIRT1-MUT) reporter vectors were constructed by integrating target fragments of wild-type and mutant SIRT1 into pGL3 vectors (Promega, Madison, WI, USA). SIRT1-WT or SIRT1-MUT was co-transfected with miR-30b-5p or negative control. After 48 hours, luciferase activity was measured. All tests were conducted in triplicate.

### RNA immunoprecipitation (RIP) analysis

RIP analysis was implemented with the EZMagna RIP RNA-binding protein immunoprecipitation kit (Millipore, Bedford, MA, USA) per the manufacturer's guidelines. The transfected HC-A cells were cracked and immunoprecipitated with the anti-human argonaute 2 (Ago2) antibody (Abcam, Cambridge, UK) and control IgG (the input group). After 48 hours of incubation, the co-precipitated RNA was separated and measured using RT-qPCR.

### Data analysis

Data in this study were processed by SPSS22.0 statistical software (SPSS Inc., Chicago, IL, USA). Three repetitive wells were set in each group, and the tests were made at least three times. Measurement data with normal distribution were represented as mean ± standard deviation (x±s). Two groups of data were compared by *t* test, and a correlation test was implemented by Pearson correlation analysis. The statistics were significant when *P*<0.05.

### Ethics statement

Our study was authorized by the Research Ethics Committee of Linyi People’s Hospital.

### Data availability statement

The data sets utilized and analyzed during the current study are available from the corresponding author on reasonable request.

## RESULTS

### miR-30b-5p was up-regulated in the joint tissues of OA patients and correlated with pro-inflammatory responses

**Figure 1:**
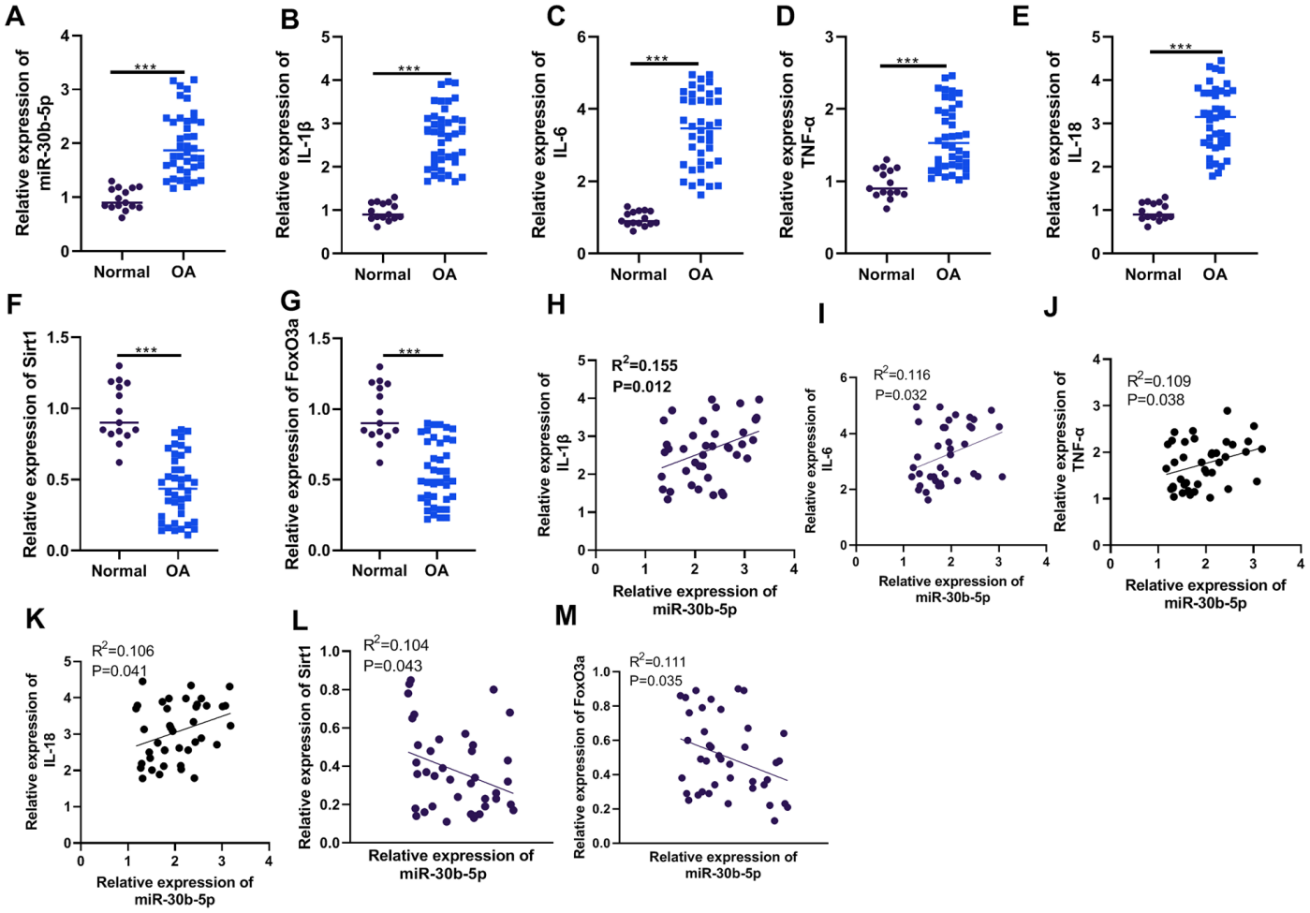
**The miR-30b-5p profile was heightened in the joint tissues of OA patients and correlated with pro-inflammatory responses.** The joint tissues of 15 non-OA patients and 40 OA patients were collected. (**A**–**G**) The levels of miR-30b-5p, IL-1β, IL-6, TNF-α, IL-18, SIRT1 and FoxO3a in joint tissues were compared by RT-qPCR. (**H**–**M**) Pearson analysis determined the correlation between miR-30b-5p, inflammatory cytokines and SIRT1/FoxO3a in OA patients’ joint tissues. ***P<0.001 (vs.Normal group).

First, we collected the joint tissues of 15 healthy donors and 40 OA patients and gauged the contents of miR-30b-5p and inflammatory cytokines (IL-1β, IL-6, TNF-α, and IL-18) in the joint tissues through RT-qPCR. The results confirmed that the miR-30b-5p profile was higher in OA patients’ joint tissues than that in normal cartilage tissues (*P*<0.05, Figure 1A). The above results also applied to inflammatory cytokines (*P*<0.05, Figure 1B–1E). Meanwhile, the profiles of SIRT1 and FoxO3a were significantly down-regulated (*P*<0.05, Figure 1F, 1G). Pearson linear correlation analysis demonstrated that miR-30b-5p was positively related to the levels of IL-1β, IL-6, TNF-α, and IL-18 in the joint tissues of OA patients (*P*<0.05, Figure 1H–1K). However, miR-30b-5p was negatively linked with the contents of SIRT1 and FoxO3a (*P*<0.05, Figure 1L, 1M). These findings signified that the miR-30b-5p profile was heightened in the joint tissues of OA patients and was positively related to the pro-inflammatory response.

### miR-30b-5p expression was facilitated in OA rat articular cartilage tissues and IL-1β-treated chondrocytes

**Figure 2:**
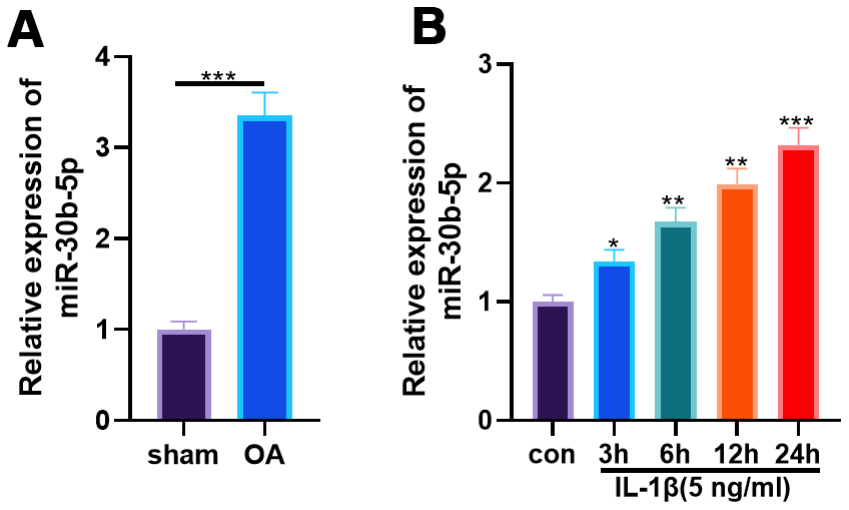
**miR-30b-5p expression was heightened in OA rat articular cartilage tissues and IL-1β-treated chondrocytes.** DMM was induced to construct an SD rat model. (**A**) The miR-30b-5p profile in OA tissues was verified by RT-qPCR, N=5. IL-1β (5 ng/mL) was adopted to treat HC-A human chondrocytes. (**B**) RT-qPCR examined the miR-30b-5p profile in HC-A cells. *P<0.05, **P<0.01, ***P<0.001, vs. con group. N=3.

We constructed an OA model in SD rats through surgical DMM and tested the miR-30b-5p level in knee tissues of SD rats. RT-qPCR results illustrated that miR-30b-5p was distinctly up-regulated in the OA group (vs. the sham group) (*P*<0.05, Figure 2A). Furthermore, HC-A cells were intervened with 5 ng/mL IL-1β for 3, 6, 12, and 24 hours, respectively. As a result, the miR-30b-5p profile was elevated in HC-A cells after IL-1β administration time-dependently (vs. the control group) (*P*<0.05, Figure 2B). These findings confirmed that miR-30b-5p was highly expressed in OA cartilage tissues and chondrocytes.

### Overexpressing miR-30b-5p intensified IL-1β-mediated chondrocyte apoptosis and inflammation

**Figure 3:**
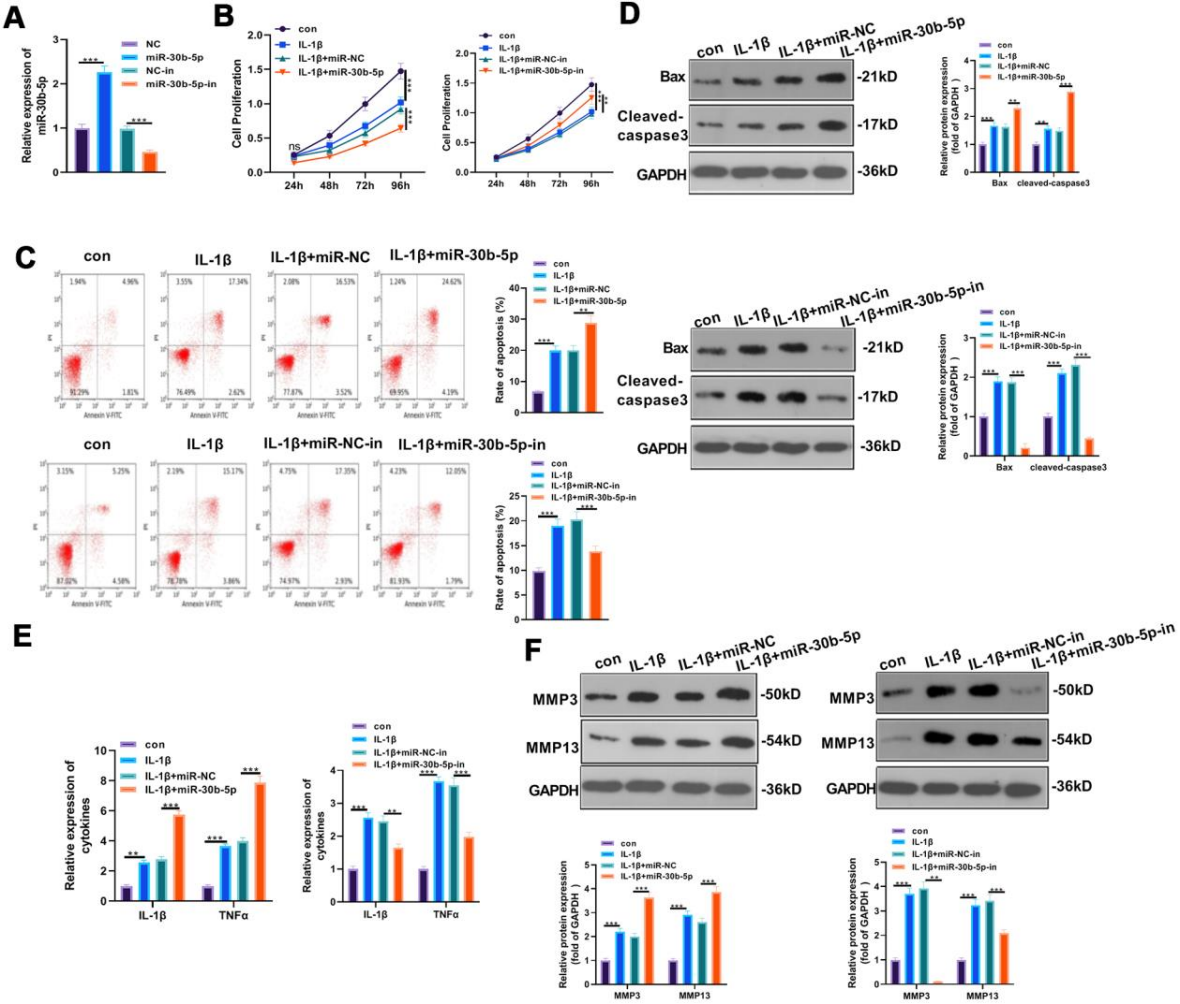
**Overexpressing miR-30b-5p aggravated IL-1β-mediated chondrocyte apoptosis and inflammation.** miR-30b-5p mimics and inhibitors were transferred into HC-A cells, respectively. (**A**) RT-qPCR verified transfection validity. (**B**, **C**) CCK8 and flow cytometry tested cell viability and apoptosis, respectively. (**D**) Profiles of Bax and Cleaved-Caspase3 were examined by WB. (**E**, **F**) The expression of IL-1β, TNFα, MMP3, and MMP13 was monitored by RT-qPCR and WB. **P<0.01, ***P<0.001. N=3.

To explore the effects of miR-30b-5p on IL-1β-induced OA, we transfected miR-30b-5p mimics and inhibitors into HC-A cells to construct miR-30b-5p overexpression and knockdown models. Meanwhile, the transfection validity was verified by RT-qPCR (*P*<0.05, Figure 3A). Cell viability was assessed by the CCK8 method, and the results manifested that HC-A cell viability was attenuated in the IL-1β group (vs. the control group). In addition, compared with the IL-1β+miR-NC group, HC-A cell viability was further hampered after miR-30b-5p mimic transfection. Nevertheless, HC-A cell viability was elevated after transfection with miR-30b-5p inhibitors (*P*<0.05, Figure 3B). Flow cytometry results showed that HC-A cell apoptosis in the IL-1β group was evidently higher than that of the control group. In contrast, compared with the IL-1β+miR-NC group, HC-A cell apoptosis was further up-regulated by miR-30b-5p mimics. On the other hand, the miR-30b-5p inhibitor transfection had the reverse function (*P*<0.05, Figure 3C). Furthermore, WB results found that the pro-apoptotic proteins Bax and Cleaved-Caspase3 were up-regulated in the IL-1β group (vs. the control group). Besides, their expression was further enhanced after miR-30b-5p mimic transfection (vs. the IL-1β+miR-NC group). On the contrary, miR-30b-5p inhibitors exerted reversed effects (*P*<0.05, Figure 3D). Moreover, the expression of IL-1β, TNF-α, MMP3 and MMP13 was compared by RT-qPCR and WB. It was found that the above-mentioned inflammatory cytokines were up-regulated in the IL-1β group (vs. the control group), and they were further up-regulated after miR-30b-5p mimic transfection (vs. the IL-1β+miR-NC group). However, the results were completely reversed after the miR-30b-5p inhibitor transfection (*P*<0.05, Figure 3E, 3F). These results illustrated that miR-30b-5p up-regulation in human chondrocytes HC-A led to significant inflammation and apoptosis.

### The impact of abating miR-30b-5p on OA rats

**Figure 4:**
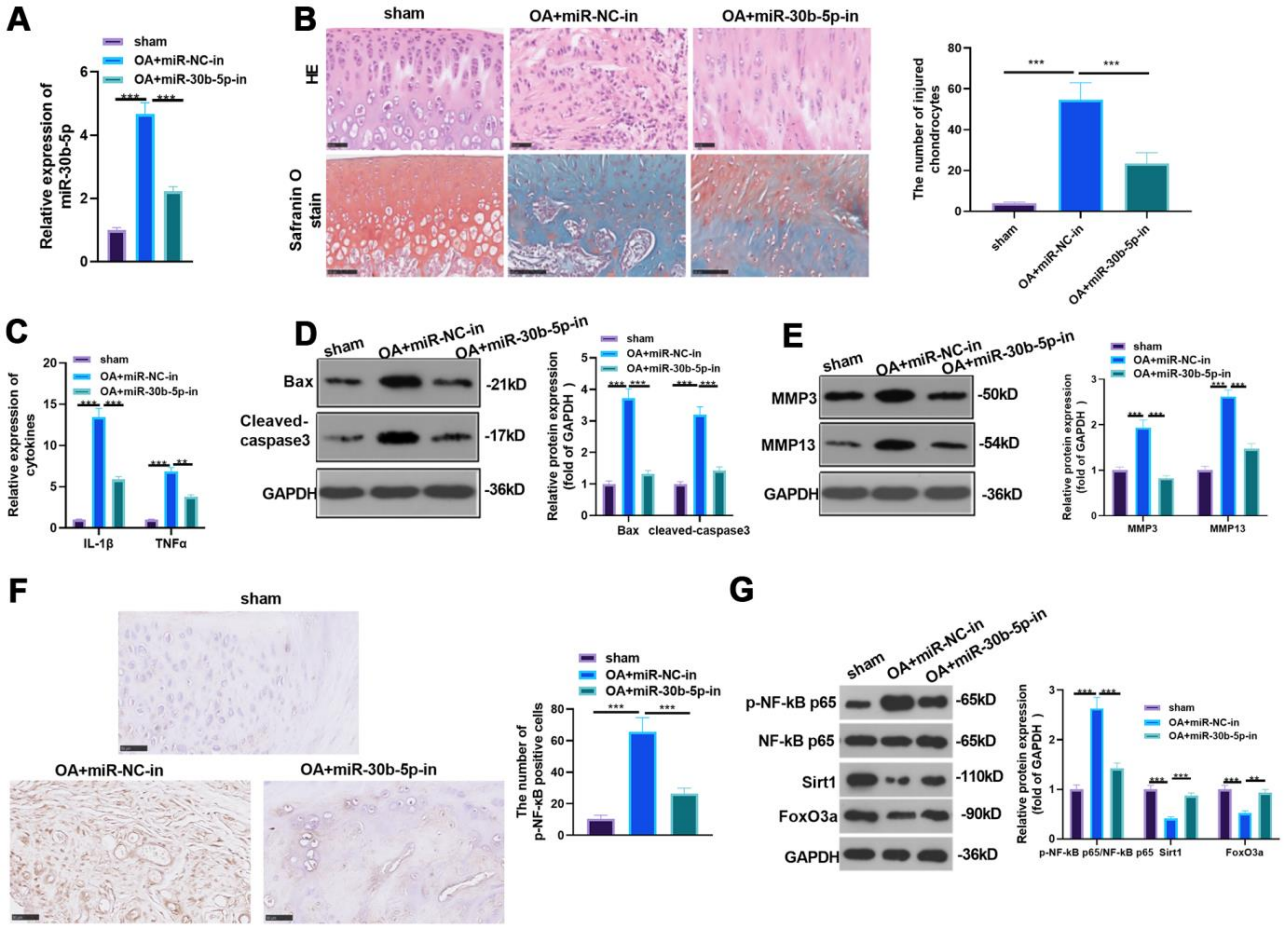
**The influence of inhibiting miR-30b-5p on OA rats.** miR-30b-5p inhibitors were added to the knee joint cavity of OA rats. (**A**) The miR-30b-5p profile was measured by RT-qPCR. (**B**) HE and Safranin O staining were used to observe the morphological differences in cartilage tissue, and the number of injured chondrocytes was counted. (**C**) The levels of IL-1β and TNF-α were compared by RT-qPCR. (**D**, **E**) WB was implemented to test the profiles of Bax, Cleaved-Caspase3, MMP3 and MMP13. (**F**) The level of NF-κB in the cartilage tissue was gauged by IHC. (**G**) Expression of NF-κB, SIRT1/FoxO3a in the cartilage tissue was compared by WB. **P<0.01, ***P<0.001. N=5.

We transfected miR-30b-5p inhibitors to the knee joint cavity and gauged the miR-30b-5p profile by RT-qPCR to probe the effect of miR-30b-5p on OA *in vivo*. It turned out that miR-30b-5p expression was curbed after the miR-30b-5p inhibitor transfection into the OA model (vs. the miR-30b-5p negative control group) (*P*<0.05, Figure 4A). Meanwhile, HE and Safranin O staining were adopted to monitor morphological deviation of rat knee joint sections. As a result, compared with the sham group, the surface articular cartilage in the OA group was worn out, and the thickness of cartilage and the bony dermal plate was reduced. After supplementing miR-30b-5p inhibitors, the above situations were improved (*P* < 0.05, Figure 4B). The contents of pro-inflammatory cytokines were further tested by RT-qPCR. The results confirmed that IL-1β and TNF-α expression in the OA group was heightened compared with that in the sham group, while they were down-regulated after the miR-30b-5p inhibitor transfection (*P*<0.05, Figure 4C). Additionally, WB data testified that Bax, Cleaved-Caspase3, MMP3 and MMP13 in OA tissues were up-regulated (vs. the sham group). Nevertheless, the above proteins in the OA+miR-30b-5p-in group were down-regulated in contrast with that in the OA+NC-in group (*P*<0.05, Figure 4D, 4E). IHC results showed that the NF-κB-positive cell number in the OA+miR-NC-in group was heightened in contrast with the sham group, while the result was opposite after miR-30b-5p inhibition (*P*<0.05, Figure 4F). The profiles of NF-κB and SIRT1/FoxO3a were further gauged by WB. As a result, the phosphorylation of NF-κB was evidently facilitated and the expression of SIRT1/FoxO3a was signally hampered in the OA+miR-NC-in group (vs. the sham group). However, the phosphorylation of NF-κB was dramatically down-regulated and the SIRT1/FoxO3a profile was markedly facilitated after miR-30b-5p inhibition (*P*<0.05, Figure 4G). These findings stated that attenuating miR-30b-5p restrained the inflammation in OA rats.

### miR-30b-5p up-regulated NLRP3 in chondrocytes and joint tissues

**Figure 5:**
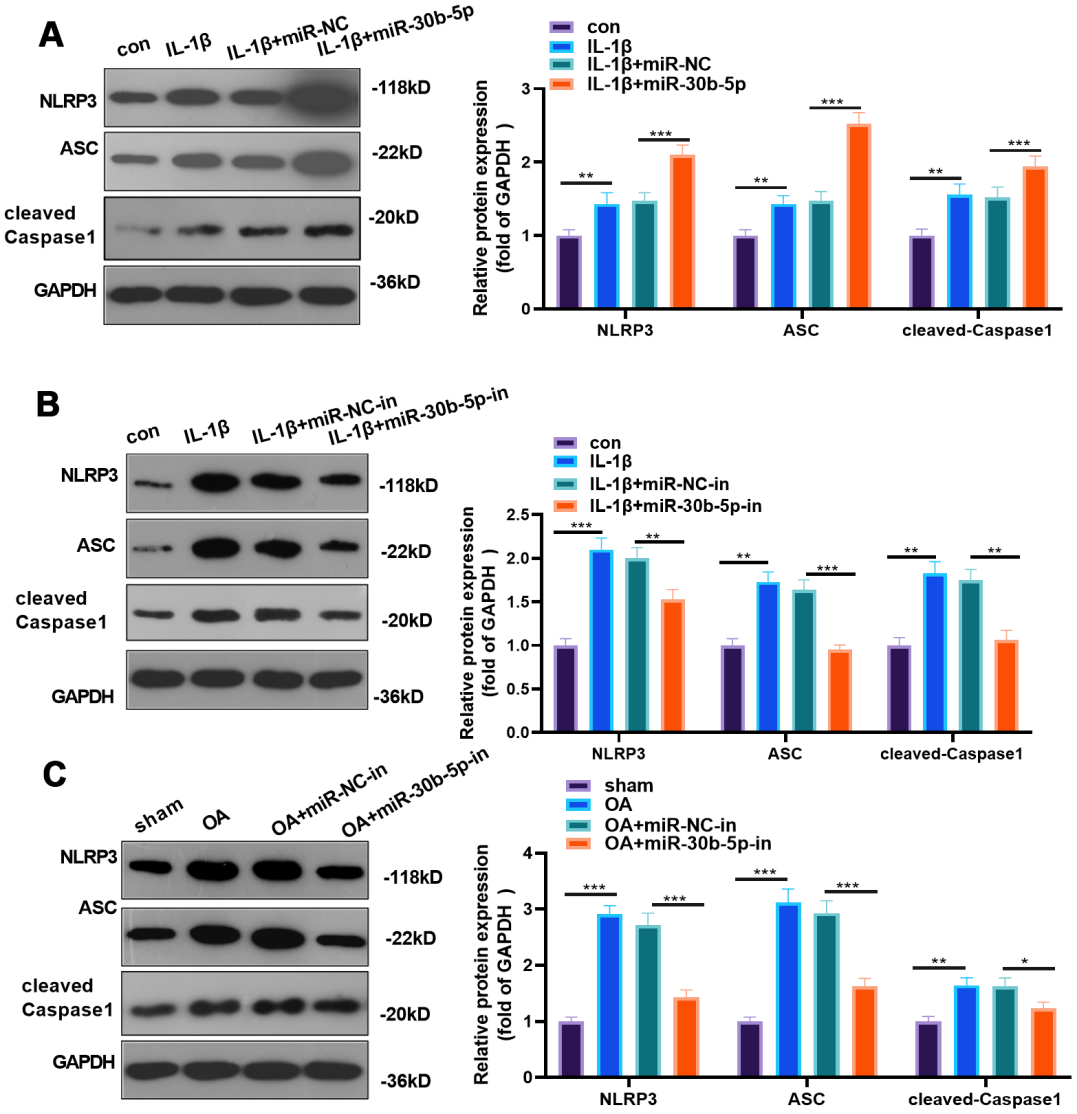
**miR-30b-5p facilitated NLRP3 expression in chondrocytes and joint tissues.** miR-30b-5p mimics or inhibitors were added to HC-A chondrocytes. (**A**, **B**) Protein expression of NLRP3-ASC-cleaved Caspase1 was verified by WB after transfecting miR-30b-5p mimics or inhibitors to IL-1β-treated HC-A cells. miR-30b-5p inhibitors were added to the knee joint cavity of OA rats. (**C**) WB examined the expression of NLRP3-ASC- cleaved Caspase1 in joint tissues after transfecting miR-30b-5p inhibitors. **P<0.01, ***P<0.001. N=5.

We adopted WB to examine the NLRP3 inflammasome expression in chondrocytes and joint tissues to probe the impact of miR-30b-5p on NLRP3. As a result, by contrast with the control group, NLRP3, ASC and cleaved-Caspase1 were significantly up-regulated in IL-1β-treated HC-A cells, and it was further up-regulated after miR-30b-5p mimics were added. On the contrary, it was down-regulated after miR-30b-5p inhibitors were supplemented (*P*<0.05, Figure 5A, 5B). Similarly, by contrast with the sham group, NLRP3, ASC and cleaved-Caspase1 were up-regulated in the OA group, while they were signally down-regulated after the supplementation of miR-30b-5p inhibitors into the knee cavity (*P*<0.05, Figure 5C). These findings revealed that miR-30b-5p up-regulated NLRP3 inflammasomes in the OA model *in vivo* and *in vitro*.

### SIRT1 targeted miR-30b-5p

**Figure 6:**
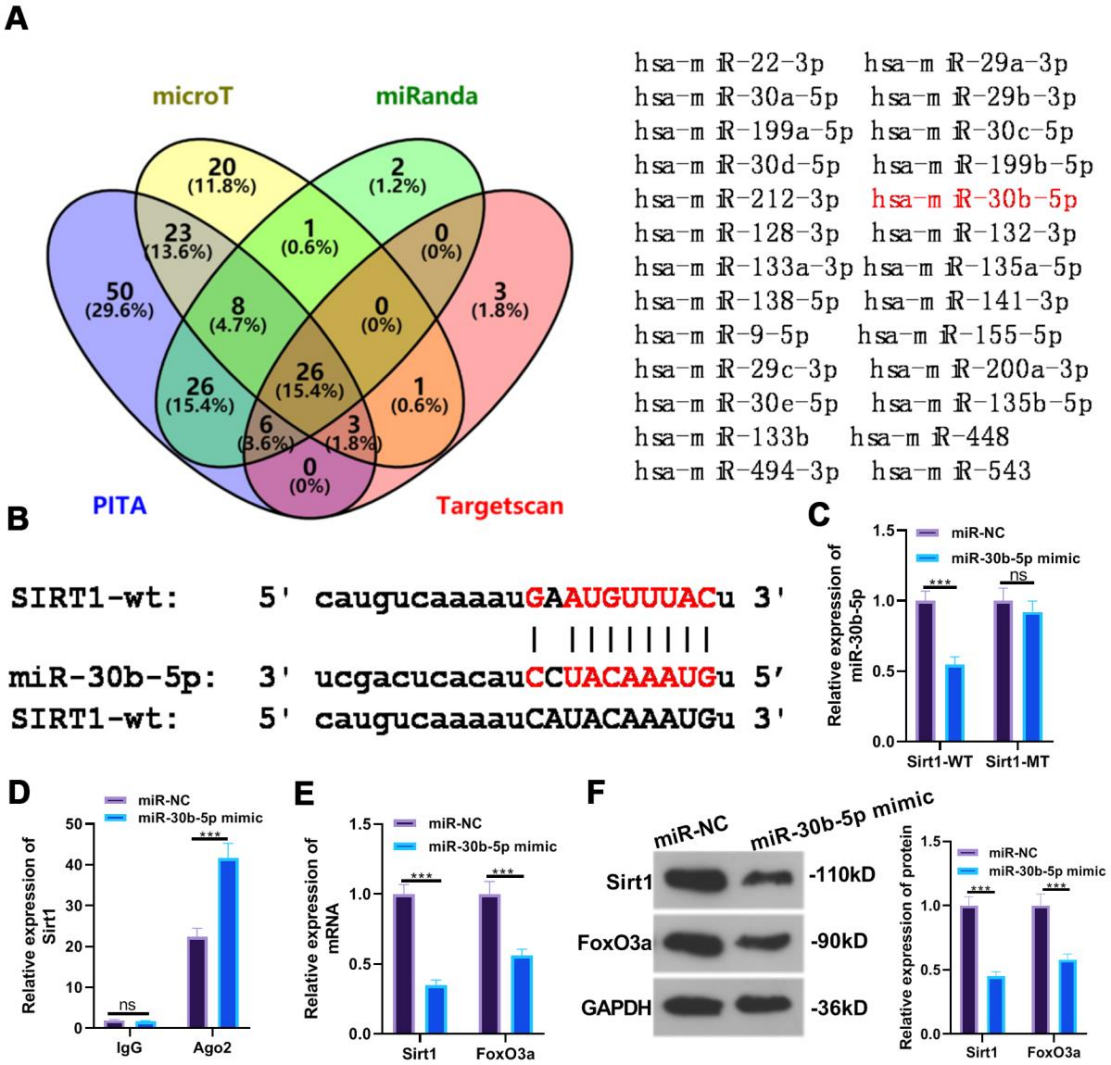
**SIRT1 targeted miR-30b-5p.** (**A, B**) The target association between miR-30b-5p and SIRT1 was predicted in the ENCORI database. (**C**, **D**) The targeted affinity between miR-30b-5p and SIRT1 was validated by the dual-luciferase reporter assay and RIP experiment, respectively. (**E**, **F**) The profiles of SIRT1 and FoxO3a were further evaluated by RT-qPCR and WB. ns*P*>0.05, ***P<0.001. N=3.

The common targets of SIRT1 were predicted via PITA, miRmap, microT and miRanda, and the shared miRNAs were validated by the Venn diagram. miR-30b-5p was discovered to be one of them (Figure 6A). Through the ENCORI database (http://starbase.sysu.edu.cn/), we discovered a binding site between miR-30b-5p and SIRT1 (Figure 6B). Furthermore, the dual-luciferase reporter assay results indicated that miR-30b-5p mimics hampered the luciferase activity of cells transfected with SIRT1-WT vectors but had little impact on that of SIRT1-MUT (*P* <0.05, Figure 6C). Additionally, RIP results illustrated that the miR-30b-5p mimic transfection led to a higher amount of SIRT1 deposited in the Ago2 antibody group than that in the IgG group, confirming that SIRT1 bound to Ago2 via miR-30b-5p (*P*<0.05, Figure 6D). The expression of SIRT1 and FoxO3a was further checked by RT-qPCR and WB, and it was found that SIRT1 and FoxO3a were evidently down-regulated after the miR-30b-5p mimic transfection (*P*<0.05, Figure 6E, 6F). These results testified that miR-30b-5p bound to SIRT1 and negatively regulated FoxO3a expression.

### Overexpressing SIRT1 weakened the miR-30b-5p-mediated effect

**Figure 7:**
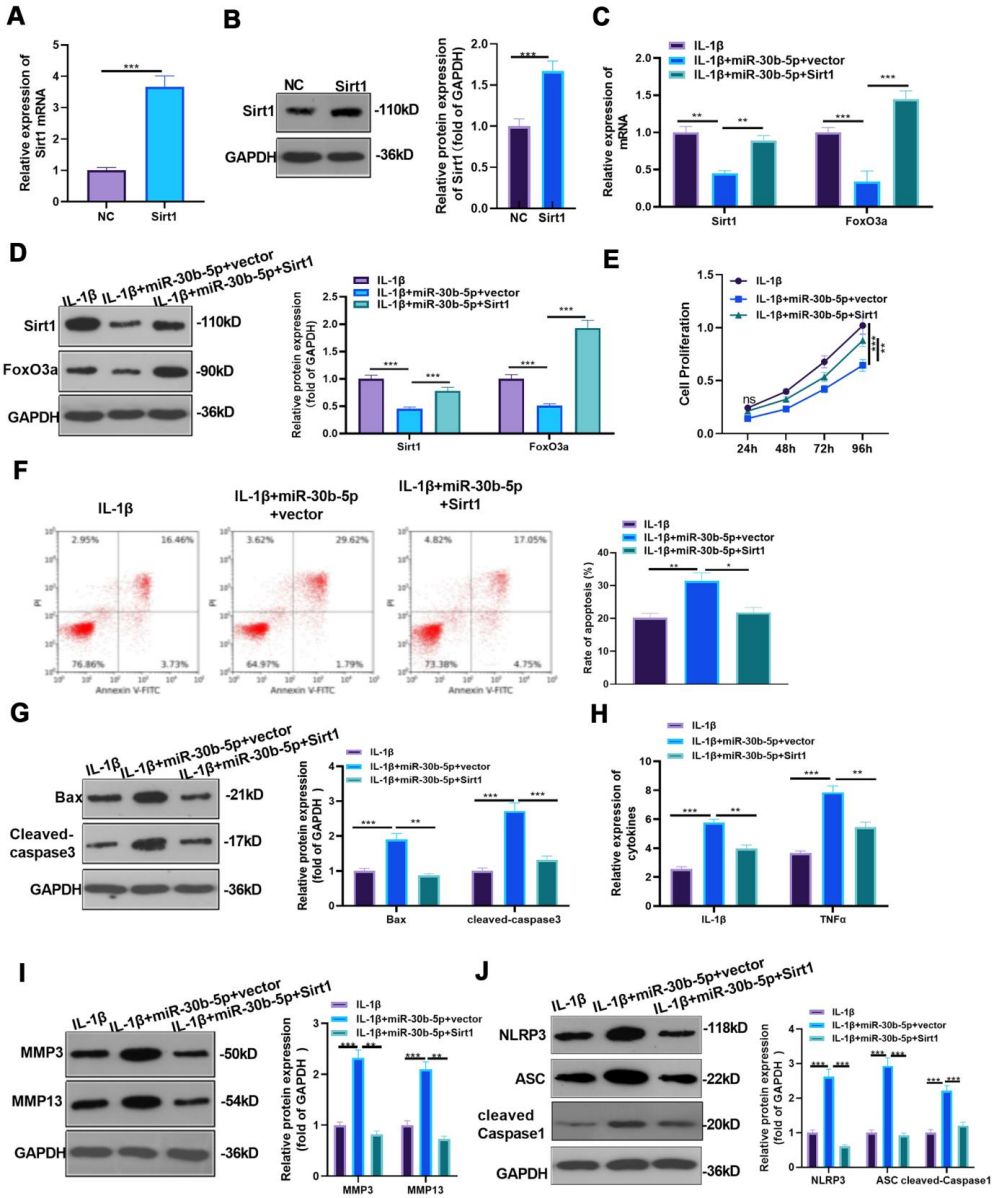
**Overexpressing SIRT1 weakened the miR-30b-5p-mediated effect.** (**A**, **B**) SIRT1 mimics were transfected into IL-1β-treated HC-A cells, and the transfection validity was verified by RT-qPCR and WB. (**C**, **D**) The expression of SIRT1 /FoxO3a in IL-1β-treated HC-A cells was assessed by RT-qPCR and WB. (**E**, **F**) CCK8 and flow cytometry monitored cell viability and apoptosis, respectively. (**G**) Profiles of Bax and Cleaved-Caspase3 were measured by WB. (**H**) RT-qPCR monitored the expression of IL-1β and TNF-α. (**I**, **J**) The profiles of MMP3, MMP13 and NLRP3-ASC- cleaved Caspase1 were verified by WB. NS*P*>0.05, *P<0.05, **P<0.01, ***P<0.001. N=3.

First, we constructed a SIRT1 overexpression model in HC-A cells and verified the transfection validity through RT-qPCR and WB to examine the influence of overexpressing SIRT1 on miR-30b-5p-mediated damaging effects (*P*<0.05, Figure 7A, 7B). Then, IL-1β was given to treat HC-A cells, and miR-30b-5p mimics were added to HC-A cells transfected with SIRT1 plasmids. The SIRT1-FoxO3a expression in HC-A cells was testified by RT-qPCR and WB. As a result, SIRT1 and FoxO3a expression was curbed in the IL-1β+miR-30b-5p+vector group (vs. the IL-1β group). Nevertheless, they were up-regulated after SIRT1 overexpression on the basis of IL-1β+miR-30b-5p+vector treatment (*P*<0.05, Figure 7C, 7D). The CCK8 assay results manifested that compared with the IL-1β group, cell viability was weakened after miR-30b-5p overexpression, while overexpressing SIRT1 on this basis promoted chondrocyte viability (*P*<0.05, Figure 7E). Besides, Flow cytometry testified that compared with the IL-1β group, overexpressing miR-30b-5p facilitated cell apoptosis, while overexpressing SIRT1 on this basis abated chondrocyte apoptosis (*P*<0.05, Figure 7F). Moreover, WB results manifested that Bax and Cleaved-Caspase3 were up-regulated after overexpressing miR-30b-5p (vs. the IL-1β group). However, compared with the IL-1β+miR-30b-5p +vector group, Bax and Cleaved-Caspase3 were significantly down-regulated in the IL-1β+miR-30b-5p+SIRT1 group (*P*<0.05, Figure 7G). Furthermore, RT-qPCR results confirmed that the contents of IL-1β and TNF-α were elevated after overexpressing miR-30b-5p (vs. the IL-1β group), while their expression was attenuated by SIRT1 overexpression on this basis (*P*<0.05, Figure 7H). Finally, WB results confirmed that MMP3, MMP13 and NLRP3-ASC-Caspase1 levels were up-regulated after overexpressing miR-30b-5p (vs, the IL-1β group). In contrast, compared with the IL-1β+miR-30b-5p+Vector group, these proteins in the IL-1β+miR-30b-5p+SIRT1 group were significantly down-regulated (*P*<0.05, Figure 7I, 7J). These experimental results signified that overexpressing SIRT1 in HC-A cells weakened the damage of miR-30b-5p on OA.

### Down-regulation of SIRT1 or FoxO3a attenuated the protection of miR-30b-5p knockdown on IL-1β-mediated HC-A cells

**Figure 8:**
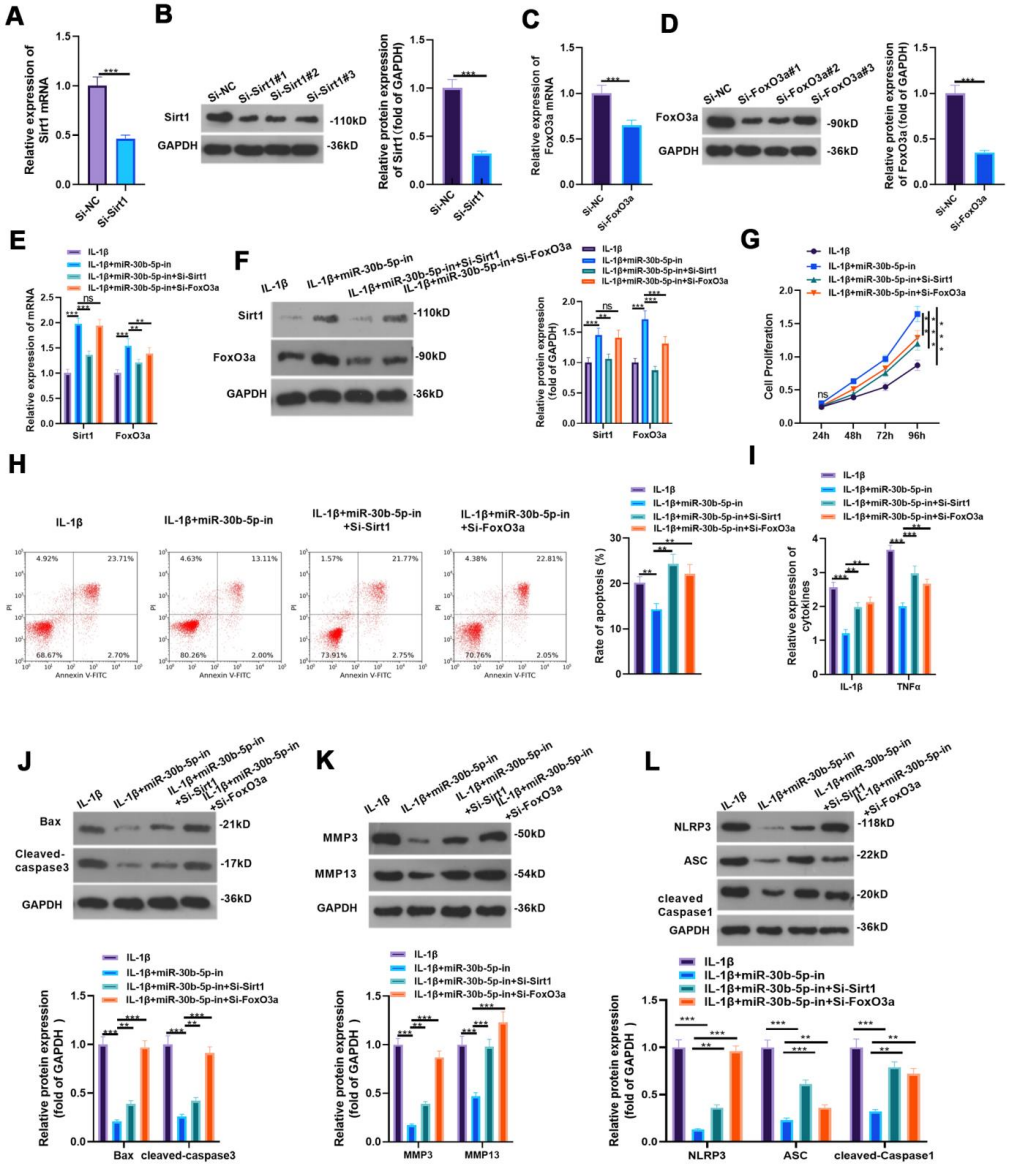
**Inhibiting SIRT1/FoxO3a curbed the protection of miR-30b-5p knockdown on IL-1β-mediated HC-A cells.** (**A**–**D**) si-SIRT1 and si-FoxO3a were transfected into IL-1β-treated HC-A cells and their transfection effects were verified by RT-qPCR and WB. (**E**, **F**) The SIRT1/FoxO3a expression in IL-1β-treated HC-A cells was checked by RT-qPCR and WB, respectively. (**G**, **H**) The CCK8 method and flow cytometry were utilized to gauge cell viability and apoptosis. (**I**) The levels of IL-1β and TNFα were monitored by RT-qPCR. (**J**–**L**) The expression of Bax, cleaved Caspase3, MMP3, MMP13 and NLRP3-ASC-cleaved Caspase1 was examined by WB. nsP>0.05, **P<0.01, ***P<0.001. N=3.

To detect the influence of inhibiting SIRT1/FoxO3a on miR-30b-5p knockdown-mediated protective effect, we constructed the low expression model of SIRT1 and FoxO3a in HC-A cells and verified the transfection effect by RT-qPCR and WB (*P*<0.05, Figure 8A–8D). Subsequently, si-SIRT1 or si-FoxO3a was respectively transfected into IL-1β-mediated HC-A cells with miR-30b-5p knockdown. As the data exhibited, SIRT1 and FoxO3a were up-regulated compared with the IL-1β+miR-30b-5p-in group. However, they were down-regulated after SIRT1 or FoxO3a knockdown (*P*<0.05, Figure 8E, 8F). CCK8 assay and flow cytometry results showed that knockdown of SIRT1 or FoxO3a impeded the activity of chondrocytes and intensified apoptosis (*P*<0.05, Figure 8G, 8H). The expression of IL-1β and TNFα was significantly decreased after miR-30b-5p inhibition (vs. the IL-1β group). On this basis, the knockdown of SIRT1 or FoxO3a strengthened the profiles of the above-mentioned inflammatory cytokines (*P*<0.05, Figure 8I). WB results testified that the expression of Bax, Cleaved Caspase3, MMP3/MMP13, and NLPRP3-ASC-Caspase1 was curbed in the IL-1β+miR-30b-5p-in group (vs. the IL-1β group). Compared with the IL-1β+miR-30b-5p-in group, the expression of the above proteins was boosted after si-SIRT1 or si-FoxO3a transfection (*P*<0.05, Figure 8J–8L). These experimental findings indicated that inhibiting SIRT1/FoxO3a in HC-A cells weakened the protective action of miR-30b-5p-in on OA.

### Inhibiting NF-κB reduced miR-30b-5p levels and IL-1β-mediated HC-A cell injury

**Figure 9:**
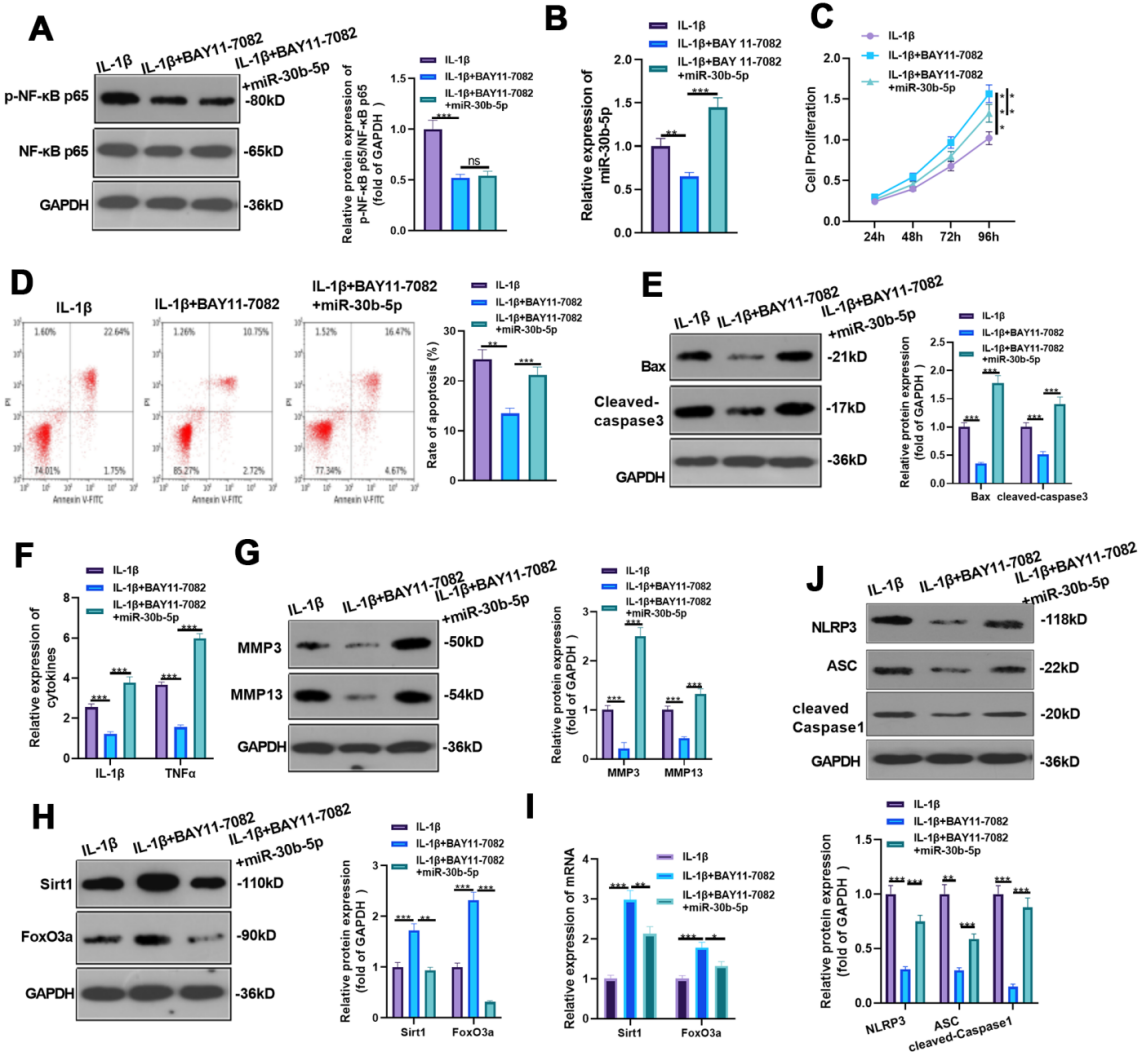
**Inhibition of NF-κB reduced miR-30b-5p levels and IL-1β-mediated HC-A cell injury.** BAY 11-7082 was added to IL-1β-mediated HC-A cells and miR-30b-5p mimics were given. (**A**, **B**) NF-κB and miR-30b-5p expression was monitored by WB and RT-qPCR, respectively. (**C**, **D**) Cell proliferation and apoptosis were determined by CCK8 and flow cytometry, respectively. (**E**) Expression of Bax and Cleaved-Caspase3 was determined by WB. (**F**, **G**) RT-qPCR and WB were conducted to compare the levels of IL-1β, TNF-α, MMP3 and MMP13. (**H**, **I**) The expression of SIRT1 and FoxO3a was assessed by RT-qPCR. (**J**) WB detected the expression of NLRP3-ASC-cleaved Caspase1 inflammasomes. **P<0.01, ***P<0.001. N=3.

We added BAY 11-7082 to IL-1β-treated HC-A cells and given miR-30b-5p mimics for intervention to verify the impact of NF-κB on the miR-30b-5p expression and IL-1β-mediated HC-A cell damage. WB and RT-qPCR were implemented to verify the profiles of NF-κB and miR-30b-5p. Interestingly, compared with the IL-1β group, NF-κB and miR-30b-5p were down-regulated after adding BAY 11-7082. However, no significant difference was observed in NF-κB expression, while miR-30b-5p was up-regulated after the miR-30b-5p mimic treatment (vs. the IL-1β+BAY 11-7082 group) (*P*<0.05, Figure 9A, 9B). Additionally, CCK8 and flow cytometry results exhibited that inhibiting NF-κB elevated HC-A cell viability and inhibited apoptosis (vs. the IL-1β group). Nevertheless, IL-1β+BAY 11-7082+miR-30b-5p weakened HC-A cell viability and strengthened cell apoptosis (vs. the IL-1β+BAY 11-7082 group) (*P*<0.05, Figure 9C, 9D). Also, WB confirmed that Bax and Cleaved-Caspase3 were down-regulated after NF-κB inhibition (vs. the IL-1β group), whereas they were significantly up-regulated in the IL-1β+BAY 11-7082+miR-30b-5p group (vs. the IL-1β+BAY 11-7082 group) (*P*<0.05, Figure 9E). The expression of IL-1β, TNF-α, MMP3 and MMP13 was compared by RT-qPCR and WB, respectively. Interestingly, their mRNA profiles were restrained in the IL-1β+BAY 11-7082 group (vs. the IL-1β group) and were up-regulated after miR-30b-5p intervention (vs. the IL-1β +BAY 11-7082 group) (*P*<0.05, Figure 9F, 9G). Furthermore, WB and RT-qPCR results uncovered that the profiles of SIRT1 and FoxO3a were up-regulated after NF-κB inhibition compared with the IL-1β group, while they were down-regulated after the miR-30b-5p mimic transfection (vs. the IL-1β+BAY 11-7082 group) (*P*<0.05, Figure 9H, 9I). WB results also manifested that the NLRP3-ASC-Caspase1 inflammasome was down-regulated after inhibiting NF-κB (vs. the IL-1β group), while it was up-regulated after adding miR-30b-5p mimics (vs. the IL-1β+BAY 11-7082 group) (*P*<0.05, Figure 9J). These findings demonstrated that inhibiting NF-κB reduced the miR-30b-5p level, and increasing miR-30b-5p significantly enhanced the injury and inflammation of HC-A cells.

## DISCUSSION

**Figure 10:**
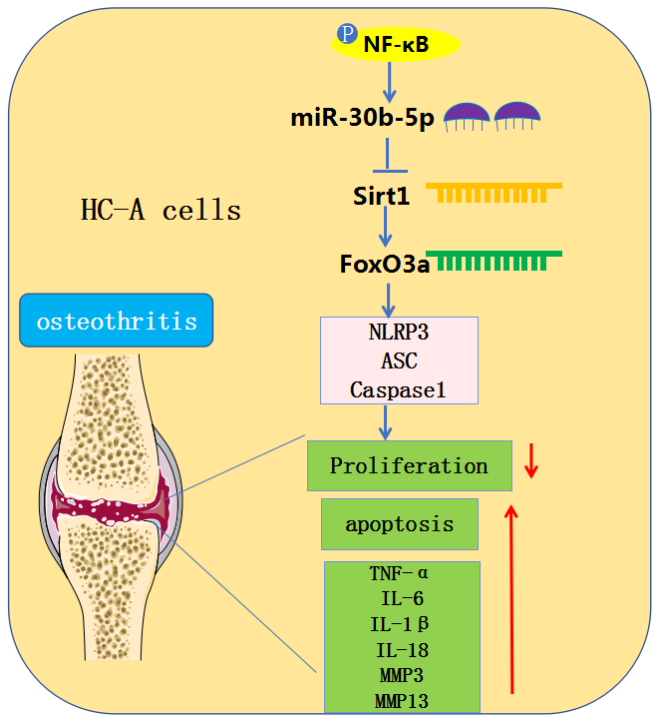
**Graphical abstract.** In OA chondrocytes, NF-κB-mediated miR-30b-5p activates NLRP3 inflammasomes by targeting and abating the SIRT1/FoxO3a expression, thereby aggravating joint pain and articular cartilage damage in OA patients.

OA is a frequent chronic orthopedic degenerative disease [22]. Due to the limited understanding of the molecular mechanism of OA, available OA treatments are limited to pain relief or joint replacement [23, 24]. A large number of studies have proved that cytokines and growth factors produced by articular cartilage under the action of mechanical and physical and chemical factors such as trauma, inflammation and infection contribute to OA pathogenesis. They are closely related to the functional changes of the synovial membrane, cartilage, and so on [25, 26]. In this study, we used surgical DMM and IL-1β to induce *in-vivo* and *in-vitro* OA models and found that overexpressing miR-30b-5p accelerated the OA articular cartilage damage by boosting the contents of IL-1β, TNF-α, MMP3, MMP13 and NLRP3 inflammasomes (Figure 10).

Previous studies have demonstrated that miR-30 contributes to tissue and organ development, tumor progression, and inflammatory injury [27]. Some studies have shown that miR-30 family members miR-30b, miR-30c, miR-30d and miR-30e are reversely related to Runx2 expression under mechanical unloading conditions, which impede osteoblast differentiation by repressing Runx2 in MC3T3-E1 cells [28]. Additionally, lncRNA MALAT1 heightens Runx2 expression and osteoblastic differentiation of fat-derived mesenchymal stem cells by targeting and dampening miR-30 [29]. miR-30b-5p is a member of the miR-30 family, which is located at 8q24.2 and 88 bp long. It is reported that miR-30b-5p exerts crucial function in myocardial hypertrophy [30], ischemic myocardial injury [31], osteosarcoma [32], bone metastasis of prostate cancer [33], and other diseases. Over the years, the function of miR-30b-5p in inflammatory diseases has also been identified. For example, Li Z et al. found that lncRNA DLEU1 up-regulates SRPK1 by binding with miR-30b-5p, which has substantial value in regulating the spinal cord inflammation in chronic constriction injury-induced rats and mediating the hypersensitivity of neuropathic pain [34]. More importantly, Wang Q et al. discovered that miR-30b-5p mediates postmenopausal osteoporosis evolvement. On the other hand, lncRNA MEG3 attenuates the osteogenic differentiation of bone marrow mesenchymal stem cells from postmenopausal osteoporosis by targeting miR-30b-5p [35]. Here, we also discovered that the miR-30b-5p profile was heightened and positively linked with pro-inflammatory responses in OA patients' articular fluid, OA rats’ articular cartilage tissues, and IL-1β-treated chondrocytes. Meanwhile, overexpressing miR-30b-5p significantly enhanced IL-1β-mediated chondrocyte apoptosis and inflammation and up-regulated IL-1β, TNF-α and NLRP3 inflammasomes. Oppositely, knocking down miR-30b-5p showed the reverse effect, which was testified *in vivo.* These findings confirmed that miR-30b-5p expedited OA evolvement.

SIRT1 is a highly conserved deacetylase dependent on NAD+ [36]. Foxo is a fork protein family subgroup, and FoxO3a is a vital member of the Foxo family. It has been found that FoxO3a expression and deacetylation are both regulated by SIRT1, and the expression and activation of the SIRT1-FoxO3a signaling pathway are involved in regulating DNA damage and repair, modulating cell differentiation, and reducing inflammation, oxidative damage, vascular diseases, and osteoporosis [37, 38]. For instance, Tseng PC et al. reported that resveratrol up-regulates RUNX2 through the SIRT1/FoxO3a axis to promote human mesenchymal stem cell osteogenesis [39]. Several studies manifested that overexpressing SIRT1 in mesenchymal stem cells facilitates the bone formation of osteoblasts through deacetylation of FoxO3a and inhibition of oxidative stress, thereby alleviating osteoporosis [40].

At the same time, Liang W et al. claimed that the SIRT1/FoxO3a axis is a potential target for osteoporosis treatment, which suggests that the SIRT1/FoxO3a contributes to orthopedic diseases [41]. Consistent with the above research, this article revealed that miR-30b-5p bound to SIRT1 and repressed its expression, and overexpressing miR-30b-5p inactivated SIRT1 and FoxO3a. Moreover, the rescue experiment illustrated that in IL-1β-treated HC-A chondrocytes, overexpressing SIRT1 significantly reduced the damage of miR-30b-5p to articular cartilage, while knocking down SIRT1 or FoxO3a reversed miR-30b-5p-in-mediated anti-inflammatory and anti-apoptosis effects. These findings confirmed that miR-30b-5p exerts its damaging effect on articular cartilage by inhibiting the SIRT1/FoxO3a expression, while overexpressing SIRT1 prevented against OA.

As one of the classical signaling pathways, TLR4/NF-κB is extensively studied in inflammation [42]. NF-κB activation has been shown to directly lead to NLRP3 inflammasome activation, and NLRP3 is of important value in the development of inflammatory diseases, especially OA [43, 44]. This article expounded that overexpressing miR-30b-5p facilitated the NLRP3 level in OA rat articular cartilage tissues and IL-1β-treated HC-A chondrocytes and mediated chondrocyte apoptosis, which was also consistent with the results of Fioravanti et al. [45]. More importantly, NF-κB activation leads to increased miR-30b-5p, while inhibition of NF-κB down-regulates miR-30b-5p, boosts HC-A cell viability, and suppresses apoptosis. Besides, miR-30b-5p targets SIRT1-FoxO3a and activates NLRP3-. This study found a new way to activate NLRP3. Namely, inhibiting NF-κB inactivates the NLRP3 pathway and alleviates OA by regulating miR-30b-5p.

Overall, this article confirmed that the miR-30b-5p profile was heightened in the articular cartilage tissues and HC-A chondrocytes of OA patients. Meanwhile, NF-κB-mediated miR-30b-5p induces NLPR3 activation by targeting the SIRT1/FoxO3a axis, thus promoting joint pain and articular cartilage damage in OA patients (Figure 10). This research offers a referable molecular mechanism for studying OA pathogenesis, while more clinical samples are needed to figure out the expression characteristics and effects of miR-30b-5p in clinical trials.
